# Etymologia: Surveillance

**DOI:** 10.3201/eid2109.ET2109

**Published:** 2015-09

**Keywords:** etymologia, surveillance, public health, Alexander Langmuir

## Surveillance [sər-vālʹəns]

From the French *surveiller*, “to watch over,” public health surveillance has its roots in 14th-century Europe. In an early form of surveillance, in approximately 1348, the Venetian Republic appointed guardians of public health to detect and exclude ships that carried plague-infected passengers. In 1662, English demographer John Graunt analyzed the mortality rolls in London and described a system to warn of the onset and spread of plague. Until the 1950s, “surveillance” referred to monitoring a person exposed to a disease; the current concept of surveillance as monitoring disease occurrence in populations was promoted by Alexander Langmuir of the Communicable Diseases Center (now the Centers for Disease Control and Prevention).

## References

[1] Declich S, Carter AO. Public health surveillance: historical origins, methods and evaluation. Bull World Health Organ. 1994;72:285–304 . 8205649 PMC2486528

[2] Dorland’s Illustrated Medical Dictionary. 32nd ed. Philadelphia: Elsevier Saunders; 2012.

